# L-Arginine Ameliorates High-Fat Diet-Induced Atherosclerosis by Downregulating miR-221

**DOI:** 10.1155/2020/4291327

**Published:** 2020-02-07

**Authors:** Hexi Zhang, Li Wang, Fei Peng, Xin Wang, Hui Gong

**Affiliations:** Department of Cardiology, Jinshan Hospital of Fudan University, Shanghai 201508, China

## Abstract

**Objectives:**

Atherosclerosis (AS) is a severe disease in which the inside of an artery narrows because of plaque formation, leading to endothelial injury in the patients. Although it has been found that endothelial nitric oxide synthase (eNOS), which produces a low concentration of NO, is necessary for endothelial function and integrity, the regulatory mechanisms of eNOS expression against the pathogenesis and development of AS are unclear. Evidence has indicated that diet supplementation with L-arginine could reduce the size of the endothelial injury lesions in AS patients. In addition, nonencoding microRNAs (miRNAs) were found to be a promising tool that regulates the expression of eNOS in human endothelial cells.

**Design:**

The aim of this research was to explore the role of L-arginine in the development of AS and the mechanisms by which miR-221 influences the possible signaling pathways in endothelial cells during AS.

**Results:**

The results suggested that L-arginine could prevent oxidized low-density lipoprotein-induced apoptosis in endothelial cells, which is associated with the downregulation of miR-221. Similar results were also observed in rat AS models.

**Conclusion:**

This research could provide potential therapies for the treatment of AS.

## 1. Introduction

Atherosclerosis (AS) is a leading cause of chronic death worldwide, and endothelial dysfunction is the first step toward coronary arteriosclerosis [1]. Endothelial injury, plaque buildup, and narrow arteries might eventually result in coronary heart disease and myocardial infarction [2]. Blood plaque, comprising of cholesterol, fat, and calcium, is responsible for limiting the flow of oxygen-rich blood to important organs, especially the heart and the brain [3]. Nitric oxide (NO) is produced by nitric oxide synthases (NOSs), which are associated with endothelial NOSs (eNOSs), neural NOSs (nNOSs), and inducible NOSs (iNOSs) [4]. The expression of eNOS, which produces a low concentration of NO, is necessary for endothelial function and integrity [5]. Endothelial-derived NO is often believed to be a protective agent in a variety of diseases and has been implicated to play a protective role against AS by reducing oxidative stress, inflammation, proliferation, and platelet aggregation [6–9].

L-arginine, a semiessential amino acid, is basic for immaturity, disease, and injury of the human body [10]. As a precursor of NO, L-arginine is metabolized and regulated by a complex set of enzymes in several signaling pathways [11]. Among these enzymes, NOS was found to be responsible for metabolizing arginine into NO and L-citrulline [11]. Enhanced NO synthesis or action leads to vasodilation and improvement in cell metabolism [12]. Evidence has indicated that supplementing the diet with L-arginine together with a statin (atorvastatin) is more efficient in reducing lesion size in AS than treatment with either L-arginine or a statin alone [13].

MiRNAs, a class of highly conservative noncoding small RNAs 19–25 bp long, are capable of degenerating the target mRNA and suppressing the mRNA translation by complementary base pairing. Twenty-five miRNAs were found to be highly expressed in human endothelial cells, of which miR-222/221 could regulate the expression of eNOS proteins [14]. Regulating eNOS proteins with these miRNAs might be beneficial in the treatment of AS; however, whether L-arginine could ameliorate the development of AS and the underlying molecular mechanisms remain unclear. The aim of this study, using an AS rat model induced by a high-fat diet, was to explore the role of L-arginine in the development of AS and the mechanisms by which miR-221 could influence the possible signaling pathways in endothelial cells during AS.

## 2. Materials and Methods

### 2.1. Animal Model

Animal studies were conducted with the approval of the ethics committee for animal care at Jinshan Hospital, Fudan University, China, and in accordance with the guidelines for Animal Experiments of the Chinese Academy of Medical Sciences.

Eight-week-old male Sprague-Dawley (SD) rats purchased from Shanghai Jiesijie Laboratories Animal Technology Co., Ltd. (Shanghai, China) were randomly divided into four groups (*n* = 10 in each group). The rats were housed in a standard animal-grade room with four animals in each cage. The temperature was maintained at 20 ± 2°C, the relative humidity at 60%, and the light cycle at 12 h/d.

The control group (Group 1) rats were given standard food and drinking water. The high-fat-fed rats (Group 2), prevention group (Group 3), and statin-treated group (Group 4) were fed a high-fat diet but Group 2 did not receive treatment. The rats in Group 3 were injected with 1 g/kg/d L-arginine (Sigma-Aldrich, Inc., St. Louis, MO, USA) over 12 weeks during AS induction. The rats in Group 4 were administered 4 mg/kg/d simvastatin for 12 weeks, and the rats in groups 1 and 2 were injected with 2 mL saline solution.

### 2.2. Morphology and Histology

Animals were anesthetized with 40 mg/kg body weight 1% pentobarbital by intraperitoneal injection and were sacrificed at 24 weeks of age. Aorta abdominalis tissue was dissected and excised between the heart and the renal artery, rinsed with cold physiological saline, and fixed in 10% formalin solution for 24 h. Dissected tissues were then dehydrated through a graded series of ethanol, diaphonized with xylol, and embedded with paraplast (Leica Biosystems, Wetzlar, Germany). The histological tissues were then cut into sections 5 *µ*m thick and stained with hematoxylin and eosin (H&E). The remaining tissue samples were flash-frozen in liquid nitrogen and stored at −80°C for further study. Transverse sections of the aorta were observed under a light microscope. The thickness of the intima and tunica media was measured using ImageJ (National Institutes of Health, Bethesda, MD, USA).

### 2.3. Primary Endothelial Cell Isolation and Culture

The aortic arteries were dissected in a sterile environment and opened using microscissors after removing adipose and connective tissues. The artery intima was submerged into a small amount of type I collagenase and digested for 1 h. Endothelial cells were isolated and collected after dissociation using 0.1% trypsin and repeated centrifugation at 1000 rpm. The detached endothelial cells were cultured in complete medium containing 20% serum (Gibco, Thermo Fisher Scientific, CA, USA) for 3-4 d. The cells were passed to the next generation after reaching 90% confluence. We used the first three generations of endothelial cells for further study.

### 2.4. L-Arginine Treatment in Endothelial Cells

Endothelial cells were divided into a control blank, oxidized low-density lipoprotein (OxLDL), 5 mM L-arginine, 25 mM L-arginine, and 50 mM L-arginine groups. Except for those in the control group, the cells in all groups were treated with 100 *µ*g/mL OxLDL (Xie Sheng Biotechnology Company, Beijing, China) in a high glucose medium. L-arginine was applied to all but the control and OxLDL groups for 24 h. All the cells were then collected for further study.

### 2.5. Transfection of AntagormiRNA

AntagormiR-221 and negative control miRNA (miR-NC) were purchased from Guangzhou RiboBio Co., Ltd. (Guangzhou, China). Briefly, 50 nM antagormiR-221/miR-NC for endothelial cells was mixed with 7 *μ*L/well lipfectamine 2000 reagent in 2 mL serum free Opti-MEM medium (Cat#: 31985070, GIBCO, Thermo Fisher Scientific). The cells were transfected for 6 h, and then the medium was replaced and maintained for up to 48 h.

### 2.6. Western Blotting

Rat aorta tissue and endothelial cells were collected and subjected to disruption using an ultrasonic homogenizer in RIPA containing protease and phosphatase inhibitors (Jiangsu KeyGEN BioTECH Corp., Ltd. Nanjing, China). Protein concentrations were detected using the Pierce BCA protein assay kit (Rockford, IL, USA). Twenty micrograms of protein from the cells (8 *μ*L for animal tissue) were separated on 8% sodium dodecyl sulfate-polyacrylamide gel electrophoresis gels and transferred to polyvinylidene difluoride membranes, which was blocked using 5% nonfat milk for 1 h at room temperature. Protein samples were incubated overnight with antibodies, rabbit *β*-actin, and rabbit eNOS (Cell Signaling Technology, Beverly, MA, USA). The secondary antibody, antirabbit immunoglobulin G-horseradish peroxidase (1 : 5000) were purchased from Cell Signaling Technology (Danvers, MA, USA). Signals were detected and analyzed using the Tanon-4500 Gel Imaging System (Tanon Science and Technology Co., Ltd., Shanghai, China).

### 2.7. RNA Extraction and Quantitative Real-Time Polymerase Chain Reaction

Total RNA was extracted from the tissues or cells using Trizol (Cat# 15596026 Invitrogen, Santa Clara, CA, USA). The concentration and quality of the RNA were checked by Nanodrop 2000 (Thermo Scientific Corp., CA). Aliquots of 2 *μ*g of RNA were treated with 1 *μ*l each of RNasin (Promega Corp., Madison, WI) and 1 *μ*l DNase I (Invitrogen Corp., Carlsbad, CA) and left at room temperature for 15 minutes, followed by heat denaturation of the DNase I at 90°C for 10 minutes. Reverse transcription was conducted using a PrimeScript™ RT Reagent Kit (Cat# RR047A, TaKaRa, Japan) with specific primers as follows: *β*-actin sense primer: 5′-CCCATCTATGAGGGTTACGC; *β*-actin anti-sense primer: 5′-TTTAATGTCACGCACGATTTC; eNOS sense primer: 5′-GCACAGGAAATGTTCACCTAC; eNOS anti-sense primer: 5′-CACGATGGTGACTTTGGCTAG; miR-221 primer: 5′-CTACATTGTCTGCTGGGTTTC. Reaction conditions for cDNA synthesis were as follows: 85°C for 15 min and hold at 4°C. Amplification using quantitative real-time polymerase chain reaction (qRT-PCR) was conducted using SYBR Premix Ex Taq II (Cat# RR820A, TaKaRa). Sample reactions were conducted at least three times. Relative gene expression data were analyzed using the 2^–ΔΔCt^ method [15] in which the threshold cycle (Ct) was obtained using the 7300 Real-Time PCR System (Applied Biosystems, Foster City, CA, USA).

### 2.8. Cell Apoptosis Assay

Endothelial cell apoptosis was determined using the Annexin V fluorescein isothiocyanate (FITC)/propidium iodide (PI) apoptosis detection assay (Thermo Fisher Scientific). The cells were digested with EDTA-free trypsin, washed twice with cold phosphate buffered saline, centrifuged, and then resuspended in 100 *µ*L binding buffer. Next, 5 *µ*L Annexin V-FITC and 5 *µ*L PI stain solution were added to the 100 *µ*L cell suspension and incubated at 37°C for 15 min. The mixtures were diluted in 400 *µ*L binding buffer and analyzed within 1 h using the Fluorescence-Associated Cell Sorter Scan (FACScan, Becton Dickinson, San Jose, CA, USA).

### 2.9. Statistical Analyses

The measurement data conforming to the normal distribution are described by the mean (M) ± standard error (SD), otherwise described by the median. Multiple groups that obeyed a normal distribution and had homogeneity of variance were determined using one-way analysis of variance. For comparison between two groups, compliance with normal distribution, and homogeneity of variance, a Bonferroni correction was applied, otherwise the Kruskal-Wallis test was used. The data were analyzed using Stata 12.0 (https://www.statalist.org), and *P* < 0.05 was considered to be statistically significant.

### 2.10. Patient and Public Involvement

No patients were involved in this study.

## 3. Results

### 3.1. Morphology of Rat Aorta Tissue

**Figure 1:**
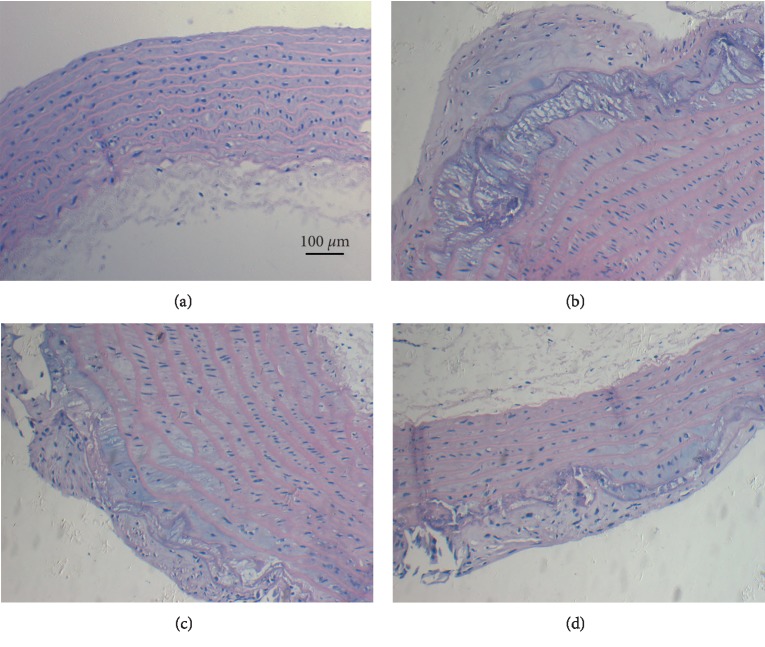
Histological morphology of rat aortic tissues. Typical hematoxylin and eosin (H&E) staining of the tissue (×100). (a) Blank group. (b) High-fat-diet group without intervention. (c) Prevention group. (d) Statin group. Scale bar is shown in Figure 1(a).

As shown in Figure 1, the tunica intimae of the aortas of the rats in the blank group were smooth and contained a small number of collagen and smooth muscle fibers, as well as uniformly distributed elastic fibers. The rats in the high-fat-diet group without intervention showed an increased number of visible foam cells and arterial wall calcification.

Rats in the L-arginine prevention and simvastatin-treated groups also showed various degrees of AS lesions in the arterial wall; however, the number of calcification lesions was significantly lower and the extent of calcification was significantly less than those in the high-fat group without intervention.  Table 1 showed that the rats in the high-fat group without intervention had the thickest tunica intimae among all the groups tested, which indicated the successful establishment of the AS rat model.

### 3.2. L-Arginine Prevents Oxidized Low-Density Lipoprotein-Induced Apoptosis in Endothelial Cells

**Figure 2:**
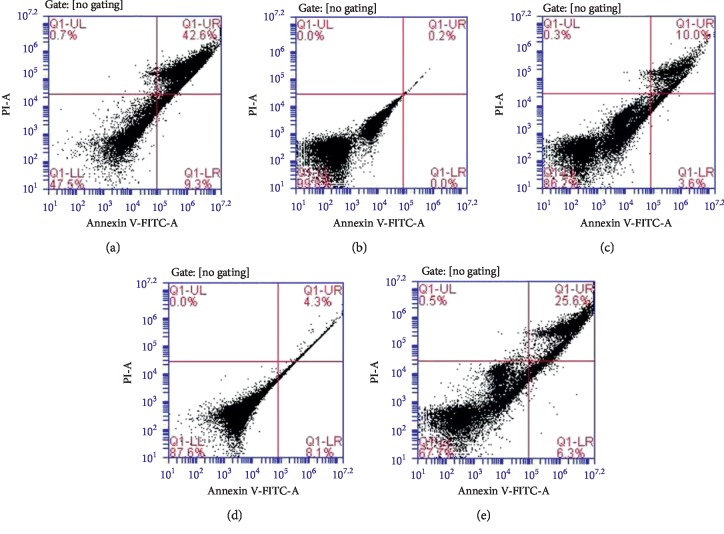
L-arginine prevents oxidized low-density lipoprotein (OxLDL)-induced apoptosis in endothelial cells. Representative FACS plots of Annexin V fluorescein isothiocyanate (FITC)/propidium iodide (PI) stained endothelial cells treated with OxLDL or cotreated with OxLDL and L-arginine at different concentrations for 24 h. (a) Blank group. (b) OxLDL-treated group. (c) 5 mM L-arginine group. (d) 25 mM L-arginine group. (e) 50 mM L-arginine group.

OxLDL could increase the permeability of endothelial cells, resulting in degeneration, shrinkage, and cell death [16]. To determine whether L-arginine can protect endothelial cells from apoptosis induced by OxLDL, we observed the apoptotic rate of endothelial cells using flow cytometry (Figure 2). The ratios of living cells after treatment of OxLDL cells with L-arginine in different concentrations were 0.2% (blank), 52.9% (5 mM L-arginine), 31.9% (25 mM L-arginine), and 12.4% (50 mM L-arginine), which suggests an eminently protective effect against OxLDL in endothelial cells.

### 3.3. L-Arginine Decreases the Expression of miR-221

**Figure 3:**
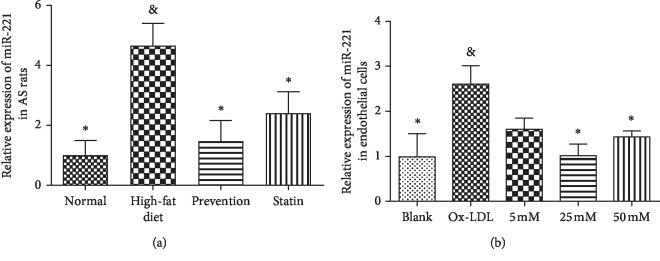
Expression of microRNA-221 (miR-221) after L-arginine treatment in atherosclerosis (AS) rat models and endothelial cells. (a) Relative expression of miR-221 and endothelial nitric oxide synthase (eNOS) in AS rat models. ^*∗*^*P* < 0.05 compared to the high-fat group; ^&^*P* < 0.05 compared to the blank group. (b) Relative expression of miR-221 in endothelial cells. ^*∗*^*P* < 0.05 compared to the oxidized low-density lipoprotein-treated group (OxLDL); ^&^*P* < 0.05 compared to the blank group. (c) Relative expression of eNOS in AS rat models. ^*∗*^*P* < 0.05 compared to the high-fat group; ^&^*P* < 0.05 compared to the blank group. (d) Relative expression of eNOS in endothelial cells. ^*∗*^*P* < 0.05 compared to the OxLDL-treated group; ^&^*P* < 0.05 compared to the blank group.

To determine whether miR-221 plays an essential role in the development of AS, qRT-PCR was used to analyze the expression of miR-221 in the AS rat model and endothelial cells. The results showed that the rats fed with a high-fat diet had a higher level of miR-221 expression than the rats in the blank group (Figure 3(a)); however, in the prevention group which was treated with L-arginine, the expression of miR-221 was decreased. An *in vitro* study showed that the OxLDL-treated endothelial cells had a higher expression of miR-221 than the blank group (Figure 3(b)). After treatment with L-arginine as the prevention drug, the expression of miR-221 decreased in OxLDL-treated endothelial cells (Figure 3(b)). Among the 5 mM, 25 mM, and 50 mM L-arginine-treated groups, the expression of miR-221 was decreased most significantly in the group treated with 25 mM L-arginine (Figure 3(b)).

### 3.4. L-Arginine Increases the Expression of eNOS

**Figure 4:**
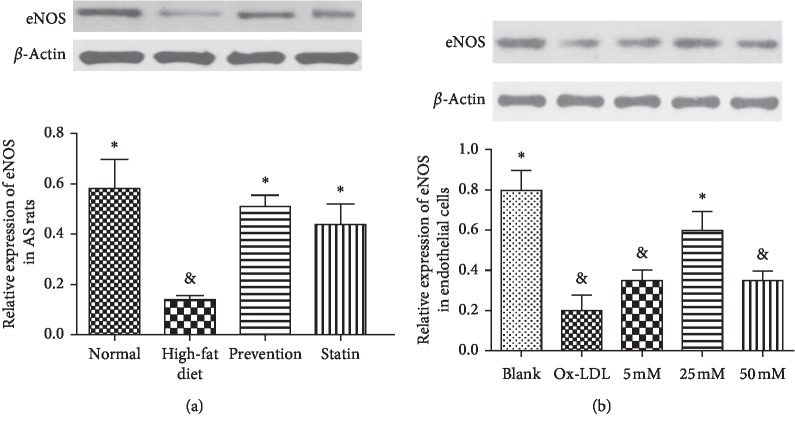
Antagomir-221 promotes the expression of endothelial nitric oxide synthase (eNOS) in endothelial cells. (a) Relative expression of eNOS mRNA after transfection with antagomir-221 or miR-NC (negative control miRNA) in endothelial cells. ^*∗*^*P* < 0.05 compared to the control; ^&^*P* < 0.05 compared to control. (b) Western blotting of eNOS in endothelial cells after transfection with antagomir-221 or miR-NC. ^*∗*^*P* < 0.05 compared to the NC; ^&^*P* < 0.05 compared to control.

In the AS rat model, the expression of eNOS in high-fat diet was significantly decreased compared to that in the blank group (Figure 4(a)). By contrast, eNOS expression in the L-arginine prevention group was increased compared to that in the high-fat-diet group (Figure 4(a)). In endothelial cells, treatment with OxLDL led to a decrease in eNOS expression compared to that in the control group (Figure 4(b), high-fat diet group vs. normal control group). However, cotreatment with L-arginine resulted in an upregulation of eNOS expression (Figure 4(b)). This increase in expression was most remarkable when the concentration of L-arginine was 25 mM (Figure 4(b)).

### 3.5. AntagomiR-221 Promotes the Expression of eNOS in Endothelial Cells

**Figure 5:**
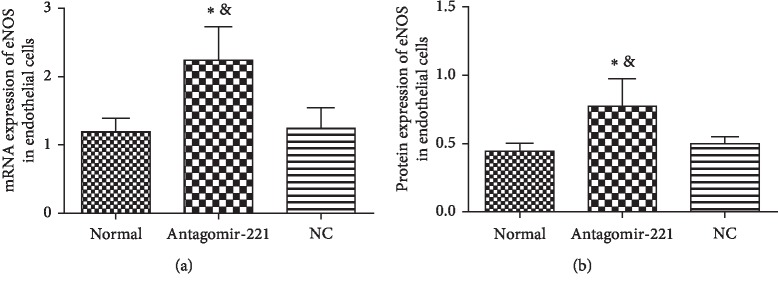
Antagomir-221 promotes the expression of eNOS in endothelial cells. (a) Relative expression of eNOS mRNA in after transfection with antagomir-221 or miR-NC (negative control miRNA) in endothelial cells. ^*∗*^*P* < 0.05 compared to the NC; ^&^*P* < 0.05 compared to control. (b) Western blot examination of eNOS in endothelial cells after transfection with antagomir-221 or miR-NC. ^*∗*^*P* < 0.05 compared to the NC; ^&^*P* < 0.05 compared to control.

To identify whether miR-221 targets eNOS and inhibits its expression, we transfected the endothelial cells with synthesized-antagomiR, the miR-221 antagonist, and investigated eNOS expression. As shown in Figure 5, the protein and mRNA levels of eNOS were significantly increased when the cells were transfected with antagomiR, which suggested that miR-221 could directly target eNOS mRNA and reduce its expression.

## 4. Discussion

In this study, the AS SD rat model was established by feeding a high-fat diet [17]. HE staining of dissected aorta tissues from the AS model rats showed classic pathogenetic morphology, such as thickening of the cell wall, obvious hyperplasia of smooth muscle, disorder of cell adherence, hyperplasia of fibrous tissue with calcification, exfoliation of endothelial cells, and thickening of the intima (intimal-medial thickness). After treatment with L-arginine, the degree of intimal-medial thickening in the prevention group was largely alleviated, and the difference between that in the prevention group and that in the control group was statistically significant. The decrease in intimal-medial thickness in the prevention group suggested that L-arginine might act as an anti-AS therapy by reducing intimal hyperplasia; however, the thickness of the middle membrane in this group was higher than that in the high-fat group, which was most likely caused by the AS process itself. The smooth muscle layer in the middle membrane becomes thickened before it atrophies during AS. L-arginine intervention delayed plaque maturation, which resulted in slower intimal-medial thickening. Accordingly, the results showed a reduction in intimal-medial thickness in the statin group, and simvastatin intervention was more obvious than that in the other groups, which may be due to the fact that there is only one concentration of L-arginine in this study. We assumed that the therapeutic effect of L-arginine would be better if the concentration is increased. On the other hand, this study showed that a high-fat diet increases intimal-medial thickness in SD rats, while L-arginine and simvastatin reduces this thickening; however, there was no obvious effect on intimal-medial thickness from the duration of drug treatment, which suggests that early anti-AS intervention with L-arginine was mainly used as protection of the vascular endothelium. Furthermore, L-arginine intervention reduced OxLDL-induced apoptosis of vascular endothelial cells *in vitro*, which also proved its protective function in the rat vascular endothelium.

We further observed an increased miR-221 expression and decreased eNOS expression in the aorta of AS rats. Similarly, in the OxLDL-treated vascular endothelial cells, we observed an increase in miR-221 expression and a decrease in eNOS expression; therefore, L-arginine could regulate the expression of miR-221 and eNOS in both rat AS models and cultured vascular endothelial cells.

Our research found that the expression levels of eNOS mRNA and eNOS protein in primary cultured rat aortic endothelial cells transfected with antagomiR-221 were significantly higher than that in the control group. The difference was statistically significant, which suggested that miR-221 can regulate eNOS expression. As indicated by Suárez et al. [17], miR-221 can regulate and increase the expression of eNOS in human umbilical vein endothelial cells by knocking out Dicer, a key enzyme in the maturation of microRNAs.

## 5. Conclusion

In this study, both L-arginine and simvastatin were found to play a protective role by inhibiting the expression of miR-221 and increasing the expression of eNOS in aortic endothelial cells of AS rats. Although the protective effect of L-arginine was weaker than that of simvastatin, it was deemed to have fewer side effects as a component of the human body, which might provide potential therapies for the treatment of AS. For future directions, the study could be expected to increase the concentration of L-arginine and apply antagomiR-221 in living high-fat diet of AS rats to see if it can reduce the increase in AS plaque and eNOS expression.

### 5.1. Limitations

The current research was only based on animal models and cell lines. More work regarding the clinical use of L-arginine on AS should be done in the future.

## Figures and Tables

**Table 1:** Thickness of the tunica media (TM) and tunica intimae (TI) in normal and high-fat-diet group rats.

Group	TI thickness (*µ*m)	TM thickness (*µ*m)
Normal	9.25 ± 0.78^*∗*^	118.82 ± 4.95^*∗*^
	105.71 ± 3.95^*&*^	150.52 ± 6.41^*&*^
Prevention	60.46 ± 2.41^*∗,&*^	174.16 ± 4.96^*∗,&*^
Statin	21.98 ± 1.35^*∗,&*^	210.21 ± 5.01^*∗,&*^

Notes: ^*&*^compared to normal control group: *P* < 0.05; ^*∗*^compared to high-fat-diet group, *P* < 0.05.

## Data Availability

The data used to support the findings of this study are available from the corresponding author upon request.

## Additional Points

L-arginine prevents oxidized low-density lipoprotein-induced apoptosis. L-arginine decreases the expression of miR-221. AntagomiR-221 promotes the expression of eNOS in endothelial cells.
